# Myocardial scar extent evaluated by cardiac magnetic resonance imaging in ICD patients: differences between polymorphic and monomorphic spontaneous events during follow-up

**DOI:** 10.1186/1532-429X-13-S1-P144

**Published:** 2011-02-02

**Authors:** Peter Bernhardt, Sascha Stiller, Daniel Walcher, Wolfgang Rottbauer

**Affiliations:** 1 University of Ulm, Ulm, Germany

## Background

Patients with ischemic cardiomyopathy have an increased risk for ventricular arrhythmia, since myocardial infarction can be the substrate for re-entrant arrhythmias. Contrast-enhanced cardiac magnetic resonance imaging (CMR) has proven to reliably quantify myocardial infarction. Aim of our study was to evaluate correlations between functional and contrast-enhanced CMR findings and spontaneous ventricular tachy-arrhythmias in patients with ischemic cardiomyopathy who underwent implantable cardioverter-defibrillator (ICD) therapy.

## Methods

Forty-one patients with ischemic cardiomyopathy and indication for ICD therapy were examined in a 1.5-T whole-body CMR system. Cine images for quantification of left ventricular volumes and function and late gadolinium enhancement images for quantification of myocardial scar extent were acquired in all patients covering the entire left ventricle.

## Results

During a follow-up period of 1184 ± 442 days 68 monomorphic and 14 polymorphic types of ventricular tachycardia (VT) could be observed in 12 patients. Patients with monomorphic VT had larger scar volumes (25.3 ± 11.3 vs. 11.8 ± 7.5 % of myocardial mass, p<0.05) than patients with polymorphic VT. Moreover myocardial infarction involved more segments in the LAD perfusion territory (86% vs. 20%, p<0.05) than in patients with polymorphic VT.

## Conclusions

Patients with spontaneous monomorphic VT during the long-term follow-up period had more infarcted tissue, which was more often present in the LAD perfusion territory than patients with polymorphic events. These data strengthen the diagnostic benefit of CMR in patients with ischemic cardiomyopathy. CMR may be used for comprehensive risk stratification in patients with ischemic cardiomyopathy undergoing ICD therapy.

